# Numerical investigation of parametric resonance due to hydrodynamic coupling in a realistic wave energy converter

**DOI:** 10.1007/s11071-020-05739-8

**Published:** 2020-06-15

**Authors:** Giuseppe Giorgi, Rui P. F. Gomes, Giovanni Bracco, Giuliana Mattiazzo

**Affiliations:** 1 Department of Mechanical and Aerospace Engineering, Politecnico di Torino, 10129 Turin, Italy; 2 IDMEC, Instituto Superior Técnico, Universidade de Lisboa, Av. Rovisco Pais 1, 1049-001 Lisbon, Portugal

**Keywords:** Parametric resonance, Parametric roll, Spar buoy, Wave energy converter, Nonlinear hydrodynamics, Floating oscillating water column

## Abstract

Representative models of the nonlinear behavior of floating platforms are essential for their successful design, especially in the emerging field of wave energy conversion where nonlinear dynamics can have substantially detrimental effects on the converter efficiency. The spar buoy, commonly used for deep-water drilling, oil and natural gas extraction and storage, as well as offshore wind and wave energy generation, is known to be prone to experience parametric resonance. In the vast majority of cases, parametric resonance is studied by means of simplified analytical models, considering only two degrees of freedom (DoFs) of archetypical geometries, while neglecting collateral complexity of ancillary systems. On the contrary, this paper implements a representative 7-DoF nonlinear hydrodynamic model of the full complexity of a realistic spar buoy wave energy converter, which is used to verify the likelihood of parametric instability, quantify the severity of the parametrically excited response and evaluate its consequences on power conversion efficiency. It is found that the numerical model agrees with expected conditions for parametric instability from simplified analytical models. The model is then used as a design tool to determine the best ballast configuration, limiting detrimental effects of parametric resonance while maximizing power conversion efficiency.

## Introduction

Spar floating platforms are axisymmetric thin and long structures that became established solutions for deep-water drilling, oil and gas extraction and storage, and, more recently, for hosting offshore wind turbines [6, 8, 27, 41]. In fact, in such applications, correct operational conditions require the floating structure to be as stable as possible, i.e., unresponsive to the wave excitation. Thanks to their reduced water-plane area and long draft, they can be designed so that their roll/pitch natural periods ($T_{n,4}=2\pi /\omega _{n,4}$) lay beyond the typical range of wave periods. However, spar buoys became popular also in the wave energy field, where the objective is to maximize the motion *just* in the degree of freedom (DoF) where the power take-off (PTO) system performs the energy conversion, while avoiding motion all others [22, 25]. In fact, response in other DoFs would effectively represent a dissipation, decreasing the power available to the PTO and, ultimately, the power efficiency [19, 21, 39].

On the one hand, a large $T_{n,4}$ makes spar buoys unresponsive in roll/pitch, hence ideal for both classic offshore applications and wave energy converters operating in heave. On the other hand, since $T_{n,4}$ is so large, $T_{n,4}/2$ typically falls in the range of operational wave periods, generating conditions for roll/pitch parametric resonance to settle [4]. Several experimental studies have confirmed the appearance of parametric roll in container ships [35], spar platforms [29] and wave energy converters (WECs), either spar buoys [7, 23] or self-reacting [5, 30, 39]. A few studies have purposely tried to exploit parametric resonance to extract energy, such as [3, 13, 43]. Conversely, for other conventional WEC concepts, parametric resonance is usually undesirable because it is often unexpected, detrimental for power extraction and threatens the device survivability. Therefore, representative mathematical models, able to accurately articulate such a nonlinear behavior, are crucial for reliable design of the mooring system [36], power-optimizing control algorithms [32, 33] and survivability strategies [19, 39]. Furthermore, only computationally fast models are eligible to be used for extensive simulations required to inform the design and control tasks.

Parametric resonance in roll is a Mathieu-type instability, arising when two conditions are met [31]: the frequency of the excitation force is about twice the natural frequency of the parametrically excited mode; the external force exceeds internal dissipations. Parametric resonance is due to nonlinear time-variations of one or more parameters of the system. In the case of a floating body, changes are due to variations of the wetted surface, determined by the relative movement of the floater with respect to the wave field. The vast majority of models for parametric resonance tend to introduce important simplifications of the system in order to fit it into an analytical framework: [34] uses multiple scale perturbation techniques for a 2-DoF model of a container ship, while [38] uses Markov and Melnikov approaches; [12] studies parametric resonance for a 2-DoF model of an archetypal spar buoy, determining nonlinear vibration modes by the application of asymptotic and Galerkin-based methods. Simplified models are successful in predicting the likelihood of parametric resonance, but are less informative about the severity of the parametrically excited response [11, 39, 42], mainly due to the mismatch between the simplified analytical model and the complex real system.

Modeling parametric resonance with analytical approaches usually requires three common but substantial simplifications about: (1) the number of DoFs, (2) the time-varying parameter and (3) the geometry. Only 2 DoFs are commonly used, although interactions between all 6 DoFs and other ancillary components (PTO, controller, mooring system, etc.) are important in generating nonlinearities and have a substantial impact on mooring loads and power production. Moreover, in order to fit into a Mathieu-type instability, it is usually assumed that the only time-varying parameter is the hydrodynamic stiffness, with simple harmonic variations. However, due to the 6-DoF motion and the complex intersection between the floater and the wave field, non-harmonic variations of both the hydrostatic stiffness and external excitation force are to be expected. Finally, archetypical geometries are usually considered, because they ease the analytical computation of main physical properties.

However, fully appreciating the nonlinear complexity of a real system is likely to require overly time-consuming models based on spatial discretization of at least the wetted surface [14, 39], or the whole fluid domain [1, 28]. Due to their computational cost, these models are unfeasible for extensive design purposes. However, this paper implements a computationally efficient nonlinear model which is able to compute in real time [17] thanks to an analytical representation of the converter wetted surface. Such a model is able to articulate parametric resonance and has been effectively used to inform the design of the mooring system of a WEC [16].

The objective of this paper is to provide a comprehensive and computationally accessible nonlinear model, able to articulate parametric resonance due to nonlinear time-variations of the parameters of the system, for a realistic device, comprising complex viscous losses, PTO, and realistic mooring system. It is shown that the model agrees with the instability conditions predicted by simplified models. Moreover, the severity of parametric resonance and the extension of the region of instability is computed, also according to a set of different physical properties of the device. In fact, since the model runs at a fraction of the computational time typically required by other analogous nonlinear models, it can be used as a design tool in order to assess the impact of parametric resonance for different control and ballast configurations.

The reminder of the paper is organized as follows: Sect. 2 introduces parametric resonance and typical analytical models, focusing on simplifications and mismatches with respect to realistic devices. Section 3 presents the device case study while Sect. 4 details the numerical model implemented. Finally, Sect. 5 discusses results and Sect. 6 presents some conclusions.

## Parametric instability

Although floating structures in unidirectional waves are externally excited only in 3 DoFs (surge, heave and pitch), under certain conditions they may respond also in the roll DoF, due to an internal excitation mechanism activated by time-variations of the system parameters. Such a phenomenon is related to parametric resonance, which is usually treated as a Mathieu-type instability [11]. The Mathieu equation is a second-order differential equation that represents the equation of motion of variable $\chi$ with the stiffness term varying harmonically over time with a frequency $\omega$ [26]:

1 $$\begin{aligned} \ddot{\chi } + \left( \varDelta +\varLambda \cos \tau \right) \chi =0, \end{aligned}$$

where $\tau =\omega t$ and the dot represent a derivative with respect to $\tau$. The parameter $\varDelta$ represents a dimensionless stiffness and $\varLambda$ is the dimensionless amplitude of the stiffness variation. In real engineering applications, the damped Mathieu equation is considered instead, which is a particular case of the Hill’s differential equation:

2 $$\begin{aligned} \ddot{\chi } + \mu \dot{\chi } + \left( \varDelta +\varLambda \cos \tau \right) \chi =0, \end{aligned}$$

where $\mu$ is the dimensionless damping coefficient.

**Fig. 1:**
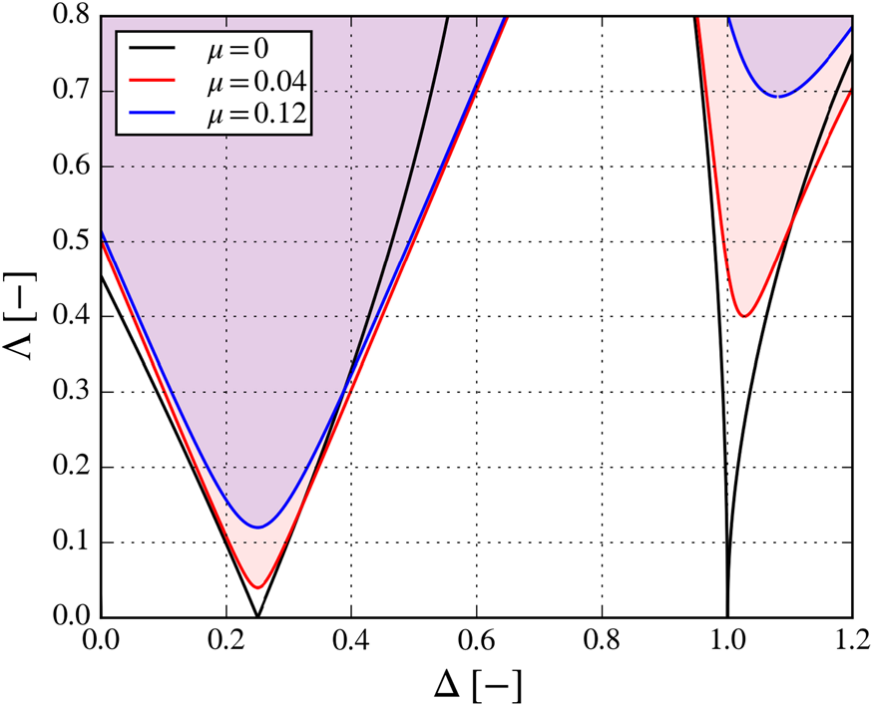
Stability diagram of the damped and un-damped Mathieu equations, shown in (1) and (2). Unstable regions are shaded

The stability diagram of equations (1) and (2) is shown in Fig. 1, where $\varDelta =\left( \omega _{n,4}/\omega \right) ^2$. Two conditions for instability (shaded areas in Fig. 1) arise:

- The excitation frequency is 2/*n* times the natural frequency of the system, with *n* being a positive integer; primary parametric resonance appears for $n=1$
- The excitation amplitude exceeds internal dissipations of the system

While the Mathieu equation can give precious insight on the conditions for parametric resonance, it is not applicable for a reliable prediction of the severity of the parametric response of a floating body, especially because there is no straightforward correspondence between the coefficient of equation (2) and the physical phenomenon. In fact, the variations of the stiffness term are, in general, not harmonic, but depend on the intersection of the floater (moving in 6 DoFs) and the wave field. Moreover, similar nonlinearities are expected in the wave excitation force and dissipations due to viscous drag. Finally, the PTO and mooring system can add further nonlinearity in 6 DoF motions.

The substantial mismatch between simplified analytical models and the physical device is discussed for a realistic case study of the spar buoy OWC (oscillating water column) WEC [22], presented in Sect. 3. A representative model, able to articulate parametric resonance, is presented in Sect. 4.

## Case study

**Fig. 2:**
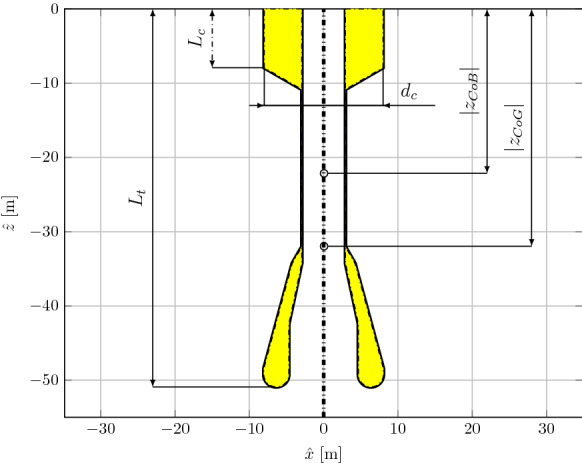
Vertical cross section of the submerged part of the body (DRT4) at equilibrium. Relevant dimensions are annotated and declared in Table 1

The spar buoy device, schematically shown in Fig. 2, is a WEC extracting energy from the relative movement between the floater and the inner water column free surface, which forces a bidirectional air flow through a turbine, acting as the PTO system. Therefore, in ideal operational conditions, pure heave movements are desirable, while any response in other DoFs would represent a decrease in the power conversion efficiency. Main geometrical and physical properties are reported in Table 1. Note that the air turbine damping effect is represented here by an equivalent orifice plate of diameter $d_0$ [23].

**Table 1:** Main physical properties of the Spar-buoy OWC device (DRT4) shown in Fig. 2, in full-scale

Parameter		Value	Units
Water depth	*h*	80.00	(m)
Diameter of the top cylinder	$d_c$	16.00	(m)
Draft of top cylinder	$L_c$	7.91	(m)
Total submerged length	$L_t$	50.91	(m)
Vertical coordinate of centre of gravity	$z_{\mathrm {CoG}}$	-31.96	(m)
Vertical coordinate of centre of buoyancy	$z_{\mathrm {CoB}}$	-22.14	(m)
Mass	*M*	$2.86\cdot 10^6$	(kg)
Perpendicular moment of inertia	$I_x=I_y$	$1.57\cdot 10^9$	(kg m$^2$)
Axial moment of inertia	$I_z$	$1.12\cdot 10^8$	(kg m$^2$)
Axial moment of inertia	$I_z$	$1.12\cdot 10^8$	(kg m$^2$)
Metacentric height	$\overline{\hbox {GM}}$	11.13	(m)
Orifice diameter	$d_o$	0.8640	(m)

Figure 2 and Table 1 refer to a configuration with draft equal to 7.91 m. However, since parametric resonance depends on inertial properties of the device, six different ballasts (hence drafts) are considered (DRT1-DRT6), as tabulated in Table 2. Since each draft configuration is characterized by a different natural period in roll ($T_{n,4}$), a shift of the parametric instability region is expected.

**Table 2:** Different draft configurations of the device, with consequent shift in natural period in roll

Conf.	*M* (kg)	$L_c$ (m)	$I_y$ (kg m$^2$)	$\overline{GM}$ (m)	$T_{n,4}$ (s)
DRT1	$2.40\cdot 10^6$	5.30	$1.39\cdot 10^9$	4.33	27.3
DRT2	$2.55\cdot 10^6$	6.17	$1.44\cdot 10^9$	6.81	23.1
DRT3	$2.71\cdot 10^6$	7.04	$1.50\cdot 10^9$	9.07	20.6
DRT4	$2.86\cdot 10^6$	7.91	$1.57\cdot 10^9$	11.13	19.0
DRT5	$3.02\cdot 10^6$	8.78	$1.64\cdot 10^9$	13.03	17.9
DRT6	$3.17\cdot 10^6$	9.65	$1.71\cdot 10^9$	14.78	17.2

## Numerical model

**Fig. 3:**
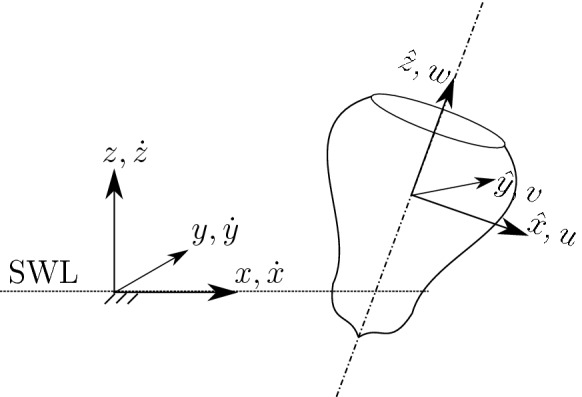
Inertial frame of reference $\left( x,y,z\right)$, centered at still water level (SWL), and body-fixed (non-inertial) frame of reference $\left( \hat{x},\hat{y},\hat{z}\right)$, after an arbitrary displacement. At rest the two frames coincide. Velocities according to the inertial frame $\left( \dot{x},\dot{y},\dot{z}\right)$ and the body-fixed frame $\left( u,v,w\right)$

The system can be studied with 7 DoFs, 6 DoFs for the floater and one additional DoF for the water column displacement. In this section, for sake of clarity and generality, the 6-DoF dynamics of the floater are first presented. It is then straightforward to expand the system to 7 DoFs. The dynamics and kinematics of the floater are conveniently represented by two right-handed frames of reference, as schematically shown in Fig. 3 for a generic axisymmetric device. The first frame $\left( x,y,z\right)$ is inertial (world-fixed), with the *x*-axis along and in the same positive direction of the wave propagation, the *z*-axis pointing upwards, with the origin at the still water level and laying on the axis of the buoy at rest. The inertial frame is used to describe the body displacements ($\varvec{\zeta }$), divided into translations ($\mathbf {p}$) and rotations ($\varvec{\varTheta }$):

3 $$\begin{aligned} \varvec{\zeta } =\left[ \begin{array}{l} \mathbf {p} \\ \varvec{\varTheta } \end{array}\right] ,\quad \mathbf {p}=\left[ \begin{array}{l} x \\ y \\ z \end{array}\right] ,\quad \varvec{\varTheta }=\left[ \begin{array}{l} \phi \\ \theta \\ \psi \end{array}\right] , \end{aligned}$$

where *x* is surge, *y* is sway, *z* is heave, $\phi$ is roll, $\theta$ is pitch, and $\psi$ is yaw.

The second right-handed frame of reference is $\left( \hat{x},\hat{y},\hat{z}\right)$, fixed with the body, hence non-inertial, and initially overlapping with the inertial frame when the buoy is at rest. The body-fixed frame is convenient for writing the dynamic equation of the system, since the inertial properties remain constant in time. Therefore, both forces and velocities are represented in the body-fixed frame, along the axis of the buoy. Velocities ($\varvec{\nu }$), divided into translation ($\mathbf {v}$) and rotations ($\varvec{\omega }$), are defined as:

4 $$\begin{aligned} \varvec{\nu } = \left[ \begin{array}{l} \mathbf {v} \\ \varvec{\omega } \end{array}\right] ,\quad \mathbf {v} =\left[ \begin{array}{l} u \\ v \\ w \end{array}\right] =\left[ \begin{array}{l} \dot{\hat{x}} \\ \dot{\hat{y}} \\ \dot{\hat{z}} \end{array}\right] , \quad \varvec{\omega }=\left[ \begin{array}{l} p \\ q \\ r \end{array}\right] . \end{aligned}$$

It is worth remarking that forces and velocities are along time-varying axes, while displacements are along fixed axes. Therefore, a mapping from body- to world-frame velocities should be applied, at each time step, in order to obtain the correct displacements. One possible mapping is the following:5where $\mathbf {R}_{\varvec{\varTheta }}$ is the rotation matrix, depending on the Euler angles $\varvec{\varTheta }$, defined according to the 3-2-1 convention as [10]:

6 $$\begin{aligned} \mathbf {R}_{\varvec{\varTheta }}&=\mathbf {R}_{\hat{z},\psi }\mathbf {R}_{\hat{y},\theta }\mathbf {R}_{\hat{x},\phi }\nonumber&\\&=\left[ \begin{matrix} c\psi &{}\quad -s\psi &{}\quad 0\\ s\psi &{}\quad c\psi &{}\quad 0\\ 0&{}\quad 0&{}\quad 1 \end{matrix}\right] \left[ \begin{matrix} c\theta &{}\quad 0&{}\quad s\theta \\ 0&{}\quad 1&{}\quad 0\\ -s\theta &{}\quad 0&{}\quad c\theta \end{matrix}\right] \left[ \begin{matrix} 1&{}\quad 0&{}\quad 0\\ 0&{}\quad c\phi &{}\quad -s\phi \\ 0&{}\quad s\phi &{}\quad c\phi \end{matrix}\right] , \end{aligned}$$

with *c* and *s* standing for $\cos ()$ and $\sin ()$ trigonometric operators, respectively. $\mathbf {R}_{\varvec{\varTheta }}$ is applied to translational velocities. $\mathbf {T}_{\varvec{\varTheta }}$ is applied to rotational ones, and is defined as follows:

7 $$\begin{aligned} \mathbf {T}_{\varvec{\varTheta }}=\left[ \begin{array}{ccc} 1&{}\quad s\phi t\theta &{}\quad c\phi t\theta \\ 0&{}\quad c\phi &{}\quad -s\phi \\ 0&{}\quad s\phi /c\theta &{}\quad c\phi /c\theta \end{array}\right] , \end{aligned}$$

where *t* stands for the $\tan ()$ trigonometric operator. Note that the singularity of $\mathbf {T}_{\varvec{\varTheta }}$ in $\pm \pi /2$ is usually not an issue in wave energy applications, since the amplitude of the pitch angle is, by design, always expected to be smaller than $\pi /2$.

Another consequences of using a body-fixed frame are Coriolis and centripetal forces, which are normally neglected under the assumption of small rotational velocities. Let us define, for convenience of notation, the skew-symmetric operator $\mathcal {S}:\mathbb {R}^3\rightarrow \mathbb {R}^{3 \times 3}$ as

8 $$\begin{aligned} \mathcal {S}:\left\{ \varvec{\lambda }\in \mathbb {R}^3 \left| \mathcal {S}(\varvec{\lambda })\overset{\varDelta }{=}\left[ \begin{array}{ccc} 0 &{}\quad -\lambda _3 &{}\quad \lambda _2\\ \lambda _3 &{}\quad 0 &{}\quad -\lambda _1\\ -\lambda _2 &{}\quad \lambda _1 &{}\quad 0 \end{array}\right] \right. \right\} . \end{aligned}$$

It follows that $\mathcal {S}(\varvec{\lambda })=-\mathcal {S}(\varvec{\lambda })^T$, and that the cross-product can be written as:

9 $$\begin{aligned} \varvec{\lambda }\times \mathbf {a}=\mathcal {S}(\varvec{\lambda })\mathbf {a} \end{aligned}$$

Using such a notation, it is possible to define Coriolis and centripetal forces as [10]:1011where *M* is the mass of the body, $\mathbf {r}_g$ is the vector from the origin of the body-fixed frame (reference point) to the centre of gravity, and $\mathbf {I}_r$ is the matrix of the moments of inertia with respect to the reference point.

Finally, the dynamical equation in 6 DoFs for the floater becomes:

12 $$\begin{aligned} {\left\{ \begin{array}{ll} \dot{\varvec{\zeta }}=\mathbf {J}_{\varvec{\varTheta }}\varvec{\nu }\\ \mathbf {M}\varvec{\dot{\nu }}+\mathbf {C}_{\mathrm{Cor}} \varvec{\nu } = \displaystyle \sum _i {\mathbf {F}}_i \end{array}\right. } \end{aligned}$$

where $\mathbf {M}$ is the inertial matrix and $\mathbf {F}_i$ comprises all external forces, namely diffraction, Froude–Krylov, radiation, drag, power take-off and mooring loads. Note that $\mathbf {F}\in \mathbb {R}^6$ is a generalized force, composed of a linear force vector $\mathbf {f}\in \mathbb {R}^3$, and a torque vector $\varvec{\tau }\in \mathbb {R}^3$. Finally, note that the 6-DoF dynamic system in (12) for the floater is readily expanded to 7-DoFs by appending the water column velocity to $\varvec{\nu }$ and expanding $\mathbf {M}$, $\mathbf {J}_{\varvec{\varTheta }}$, $\mathbf {C}_{\mathrm{Cor}}$, and $\mathbf {F}$ accordingly.

While radiation and diffraction can be assumed as linear [18, 37], a nonlinear representation of FK forces, viscous drag effects, PTO force, and mooring loads is implemented, as further explained in following subsections.

### PTO force

The power take-off system is an air turbine, which converts the alternating air flow induced by the water column motion relative to the floater. The pressure drop across the turbine can be simulated using an orifice plate which, neglecting compressibility [9], induces a PTO force of:

13 $$\begin{aligned} F_{\mathrm{PTO}} = \frac{8\rho _a A_a^3}{\pi ^2 C_d^2 d_0^4}\left( \dot{\hat{z}} - \dot{\hat{z}}_7 \right) \left| \dot{\hat{z}} - \dot{\hat{z}}_7 \right| \end{aligned}$$

where $\rho _a$ is the air density, $A_a$ is the cross-sectional area of the air chamber, $C_d$ is the discharge coefficient ($C_d=0.6466$ [23]), $d_0$ is the diameter of the orifice, and $\dot{\hat{z}}_7$ is the velocity of the water column along the axis of the buoy. Note that $F_{\mathrm{PTO}}$ acts on both the buoy and the water column, but with opposite sign.

The damping introduced to the system by the PTO, depending on the area of the orifice opening, is a control parameter that can be used to maximize the power extraction, as well as hinder the development of parametric resonance. Therefore, the sensitivity of the parametric roll amplitude and power conversion efficiency to different $d_0$ configurations has been studied. Diameters in Table 3 are considered, including 4 operational conditions with the areal ratio between orifice and water column between 0.65% and 4.31%, one almost-closed condition, with areal ratio of 0.10% that effectively makes the water column and floater move together, and a free-flow condition, with areal ratio of 20% that makes the floater and the water column move independently.

Note that the closed and free-flow conditions are often alternative solutions in survivability strategies in severe wave conditions, when avoiding failures acquires higher priority than producing power. Models that articulate parametric resonance are crucial in such analysis, since parametric roll, potentially threatening the device survival, depends on the damping and stiffness characteristics of the system, which are modified by the PTO force [39].

**Table 3:** Different orifice diameters and areas, and areal ratio with respect to the area of the water column

$d_0$ (m)	Area (${\hbox {m}}^2$)	Areal ratio (%)	
0.1863	0.0273	0.10	($\approx$ closed)
0.4739	0.1764	0.65	
0.6968	0.3813	1.40	
0.8640	0.5863	2.15	
1.2218	1.1724	4.31	
2.6351	5.4536	20.0	($\approx$ free-flow)

### Mooring force

**Fig. 4:**
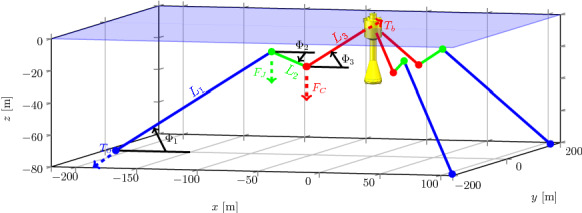
Schematics of the mooring system layout for configuration DRT4, with three lines $120^\circ$ apart. Each line comprises an anchor, a jumper, and a clump-weight. Figure modified from [16]

The mooring system, schematically shown in Fig. 4, is based on experiments performed in Plymouth, UK [7]. It is composed of three lines, equally spaced in the radial direction around the vertical axis of the buoy at rest. Each line is divided in three segments, connecting the anchor to a jumper (line of length $L_1$), then to a clump weight (line of length $L_2$), and finally to the buoy (line of length $L_3$). Relevant parameters for the equivalent full-scale model of the mooring system are tabulated in Table 4.

**Table 4:** Parameters of the full-scale mooring system for configuration DRT4, based on the experimental tests in [7]

Parameter		Value	Units
Line diameter	$d_l$	32	(mm)
Net line density	$\rho _L^*$	3.55	(kg/m$^{3}$)
Jumper mass	$M_J$	4030.5	(kg)
Jumper density	$\rho _J$	123.00	(kg/m$^{3}$)
Jumper mass	$M_C$	36140	(kg)
Clump-weight density	$\rho _C$	8097.50	(kg/m$^{3}$)
Length anchor $\rightarrow$ jumper	$L_1$	143.28	(m)
Length jumper $\rightarrow$ clump-weight	$L_2$	37.01	(m)
Length clump-weight $\rightarrow$ buoy	$L_3$	50.40	(m)
Radius at the anchor	$R_a$	211.2	(m)
Depth at the anchor	*h*	80	(m)
Attachment radius at the buoy	$R_b$	$-9.28$	(m)
Attachment depth at the buoy	$h_b$	$-2.58$	(m)

A quasi-static model is defined to compute the tension on each line depending on the 6-DoFs displacements of the attachment points of the buoy and consequently obtain the total forces and torques acting on the floater, around the origin of the body-fixed frame and along its axes. Relying on the fact that for this system each line has a relatively high tension when compared with their mass, it is possible to model each mooring line segment as a rigid and inelastic line. Consequently, for each line, two equations are written for the vertical and horizontal force equilibrium, one for the torque balance, and two for imposing geometrical constraints [16].

### Nonlinear hydrodynamic forces

The main source of time variations of system parameters inducing parametric instability are nonlinear Froude–Krylov forces [39], which are the integral of the undisturbed pressure field onto the *instantaneous* (time-varying) wetted surface ($S_w(t)$) of the floater:

14a $$\begin{aligned}&\mathbf {f}_{FK}=\mathbf {f}_g+ \iint \limits _{S_w(t)} P \mathbf {n} \ \mathrm{d}S, \end{aligned}$$

14b $$\begin{aligned}&\varvec{\tau }_{FK}=\mathbf {r}_g\times \mathbf {f}_g+ \iint \limits _{S_w(t)} P \mathbf {r}\times \mathbf {n} \ \mathrm{d}S, \end{aligned}$$

where *P* is the pressure field, $\mathbf {f}_g$ is the gravity force, $\mathbf {n}$ is the unity vector normal to the surface, $\mathbf {r}$ is the generic position vector, and $\mathbf {r}_g$ is the position vector of the centre of gravity. For geometries of arbitrary complexity, it is necessary to perform a spatial discretization of the wetted surface by means of plane mesh panels [14], implying the use of a computationally expensive re-meshing routine to recompute, at each time step, the submerged portion of the device. However, for axisymmetric geometries as spars, a convenient analytical representation of the wetted surface can be defined, using cylindrical coordinates $\left( \varrho ,\vartheta \right)$ in the body-fixed frame. The integral in (14a), for example, after appropriate mapping from inertial frame to body-fixed frame, becomes [16]:

15 $$\begin{aligned} \begin{aligned} \mathbf {f}_{FK}&= \mathbf {R}_{\varvec{\varTheta }}^T\mathbf {f}_g + \iint \limits _{S_w(t)}P(\hat{x},\hat{y},\hat{z}) \ \mathbf {n} \ \mathrm{d}S\\&= \mathbf {R}_{\varvec{\varTheta }}^T\mathbf {f}_g + \int \limits _{-\pi }^{\pi }\int \limits _{\varrho _1}^{\varrho _2}P(\varrho ,\vartheta )\left( \mathbf {e}_\varrho \times \mathbf {e}_\vartheta \right) \ \mathrm{d}\varrho \ \mathrm{d}\vartheta , \end{aligned} \end{aligned}$$

The analytical description of the instantaneous wetted surface, hence the integrals in (15), enables computation in about real time and, therefore, extensive sensitivity analysis and design optimization [16]. Parametric coupling is mainly due to nonlinear Froude–Krylov forces, shown in (15). This can be verified by inspection of the mathematical structure of an analytical representation provided in [24], obtained thanks to multivariate Taylor expansion. The formulation in (15) is notionally equivalent to the one in [24], relying on direct numerical integration instead of series expansion.

A further source of nonlinearity is the viscous drag force, acting in all DoFs with a notional quadratic dependence on the relative velocity between the floater displacement and the fluid velocity field. Due to the typically long draft of a spar, an integral formulation is adopted, using the same coordinates as in (15) [16].

**Fig. 5:**
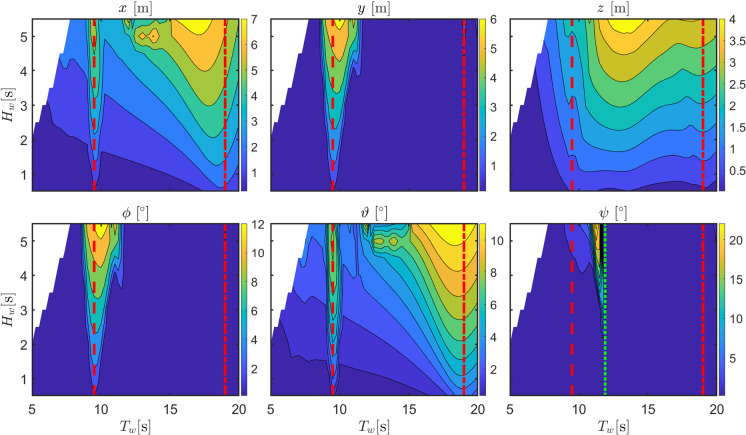
Motion amplitude of the six DoF of the floater DRT4 for the optimal orifice diameter. Dashed and dash-dotted red lines are at $T_{n,4}/2$ and $T_{n,4}$, respectively. The dashed green line is at $T_{n,6}/2$

## Results

A refined set of representative regular waves is considered, with period ($T_w\in \left[ 5\,\text {s},20\,\text {s}\right]$) and height ($H_w\in \left[ 0.5\,\text {m},5.5\,\text {m}\right]$) in the typical range of operation. However, waves with excessive steepness (higher than 6%) are excluded from the analysis due to physical constraints of the linear potential flow theory. Figure 5 shows an example of the amplitude of the displacements in 6 DoFs of the floater (configuration DRT4), with the orifice diameter that maximizes power production. Dashed and dash-dotted red lines highlight $T_{n,4}/2$ and $T_{n,4}$, respectively.

As expected, parametric resonance produces a roll response in the vicinity of $T_{n,4}/2$, with the instability region widening as the wave height increases, with a consequent increase in the amplitude of oscillation. The motion in sway is induced by the coupling with roll due to mooring and hydrodynamic restoring forces. Since, in axisymmetric floaters, the natural period in pitch ($T_{n,5}$) is the same as in roll, also pitch is prone to experience parametric instability. In fact, Fig. 5 shows a clear local increase in pitch around $T_{n,4}/2$. Similarly to the sway-roll pair, also surge is coupled with pitch. Finally, note that also the yaw DoF shows a local response only around $T_{n,6}/2$, due to parametric instability induced by a nonlinear stiffness effect in the tangential direction of the mooring lines at the fairleads [16].

**Fig. 6:**
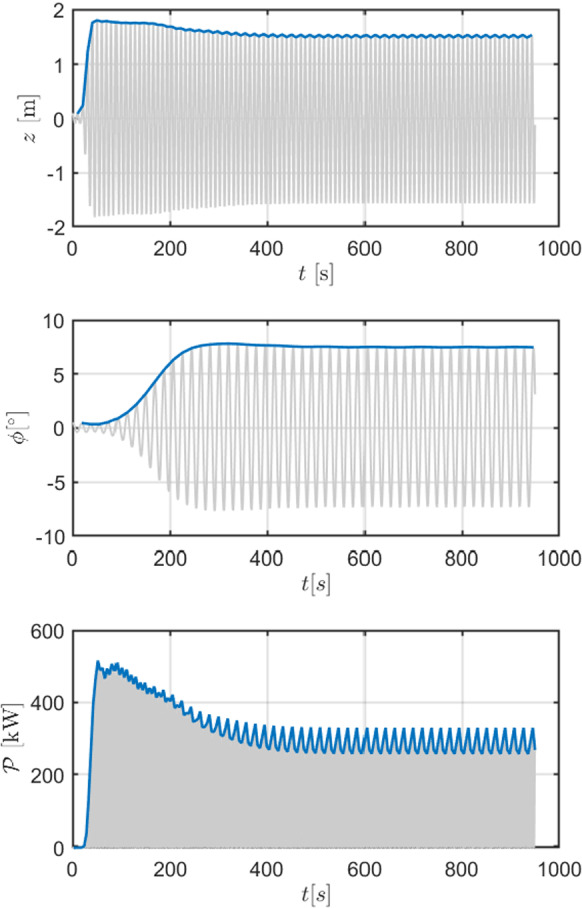
Example of time trace (in grey) and envelope (in blue) for heave (top), roll (middle) and generated power (bottom) for configuration DRT4, with $d_0=0.864$ m, in parametric resonance conditions ($T_w=T_{n,4}/2= 9.5$ s and $H_w=3$ m). (Color figure online)

The increase in surge, sway, roll and pitch DoFs is the main reason why parametric resonance has significant impact on the device survivability and design of the mooring system. On the other hand, it is possible to notice a local drop of heave response when parametric roll appears. In fact, since parametric instability opens a channel to internally transfer energy from heave to other DoFs, the power available to the PTO and the conversion efficiency decreases. This process is particularly evident in the time traces and envelope shown in Fig. 6. Since parametric roll response has a significantly longer transient than externally excited DoFs, it is possible to remark the energy transfer from heave to roll, making the heave displacement decrease as roll increases. Despite the fact that the drop in heave amplitude is apparently small, the generated power experiences a significant decrease, showing how detrimental parametric resonance is for energy absorption and conversion.

**Fig. 7:**
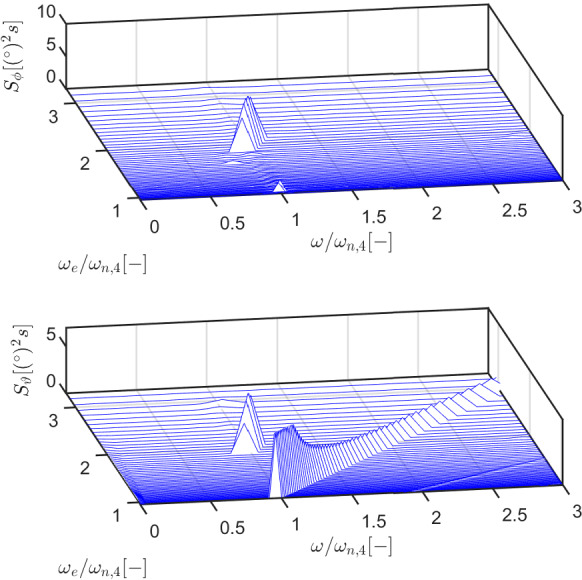
Example of waterfall plot for roll (top) and pitch (bottom) for configuration DRT4, with $d_0=0.864$ m and $H_w=3$ m. The corresponding map is shown in Fig. 8. A waterfall plot represents a series of fast-Fourier transforms for different excitation frequencies ($\omega _e$). Frequencies in the horizontal axis are normalized by the natural frequency in roll ($\omega _{n,4}$)

**Fig. 8:**
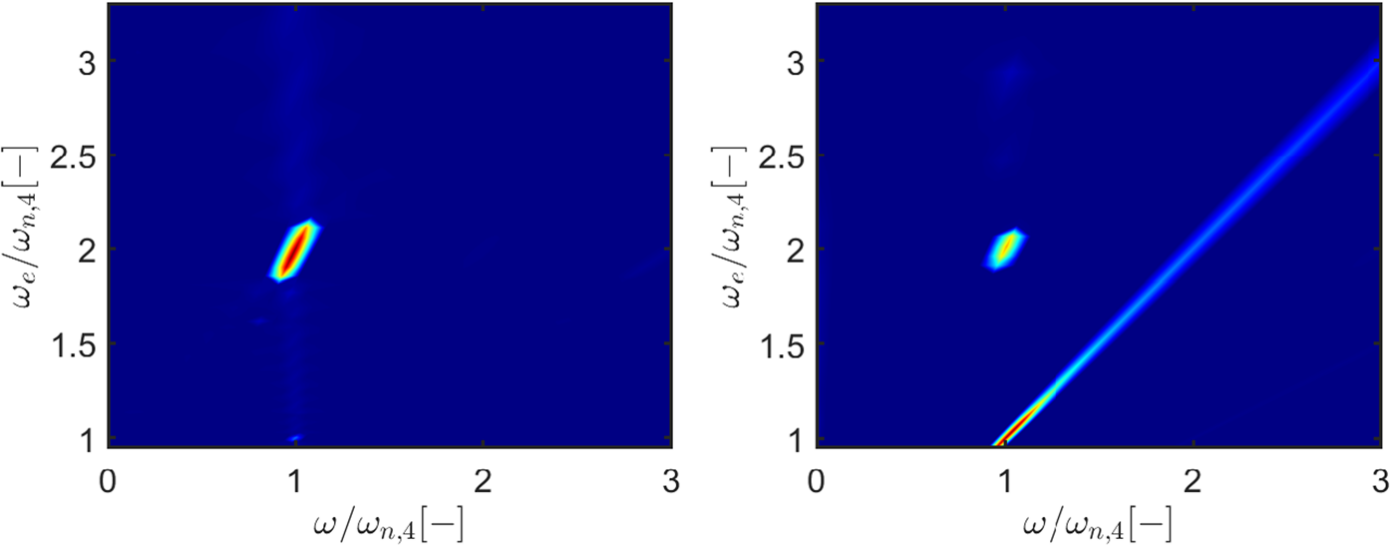
Example of waterfall colour-map for roll (top) and pitch (bottom) for configuration DRT4, with $d_0=0.864$ m and $H_w=3$ m. The corresponding plot is shown in Fig. 7. A waterfall map represents a series of fast-Fourier transforms for different excitation frequencies ($\omega _e$). Frequencies in the horizontal axis are normalized by the natural frequency in roll ($\omega _{n,4}$)

Figures 5 and 6 show that parametric resonance is not just evident in roll, which is excited only internally, but also in heave and pitch, that show a clear perturbation at the resonance instability frequency as parametric resonance arises. The waterfall plots and maps in Figs. 7 and 8 represent the fast Fourier transform (FFT) for different incoming wave frequencies $\omega _e$, at a constant wave height ($H_w=3$m). All frequencies are normalized by the natural period in roll/pitch. All the spectral energy in roll is focused around $\omega /\omega _{n,4}=1$ and only when $\omega _e/\omega _{n,4}=2$, generating roll oscillations at a frequency which is half the excitation frequency.

The spectral energy in the pitch DoF is divided into two regions. Similarly to roll, parametric pitch generates a response at $\omega /\omega _{n,4}=1$ when $\omega _e/\omega _{n,4}=2$. Parametric pitch is superimposed to the linear behavior that makes the floater pitch at the same frequency of the excitation force. This is particularly evident in the map in Fig. 8, since the spectral energy lays on the bisector of the plane, i.e., at $\omega =\omega _e$.

**Fig. 9:**
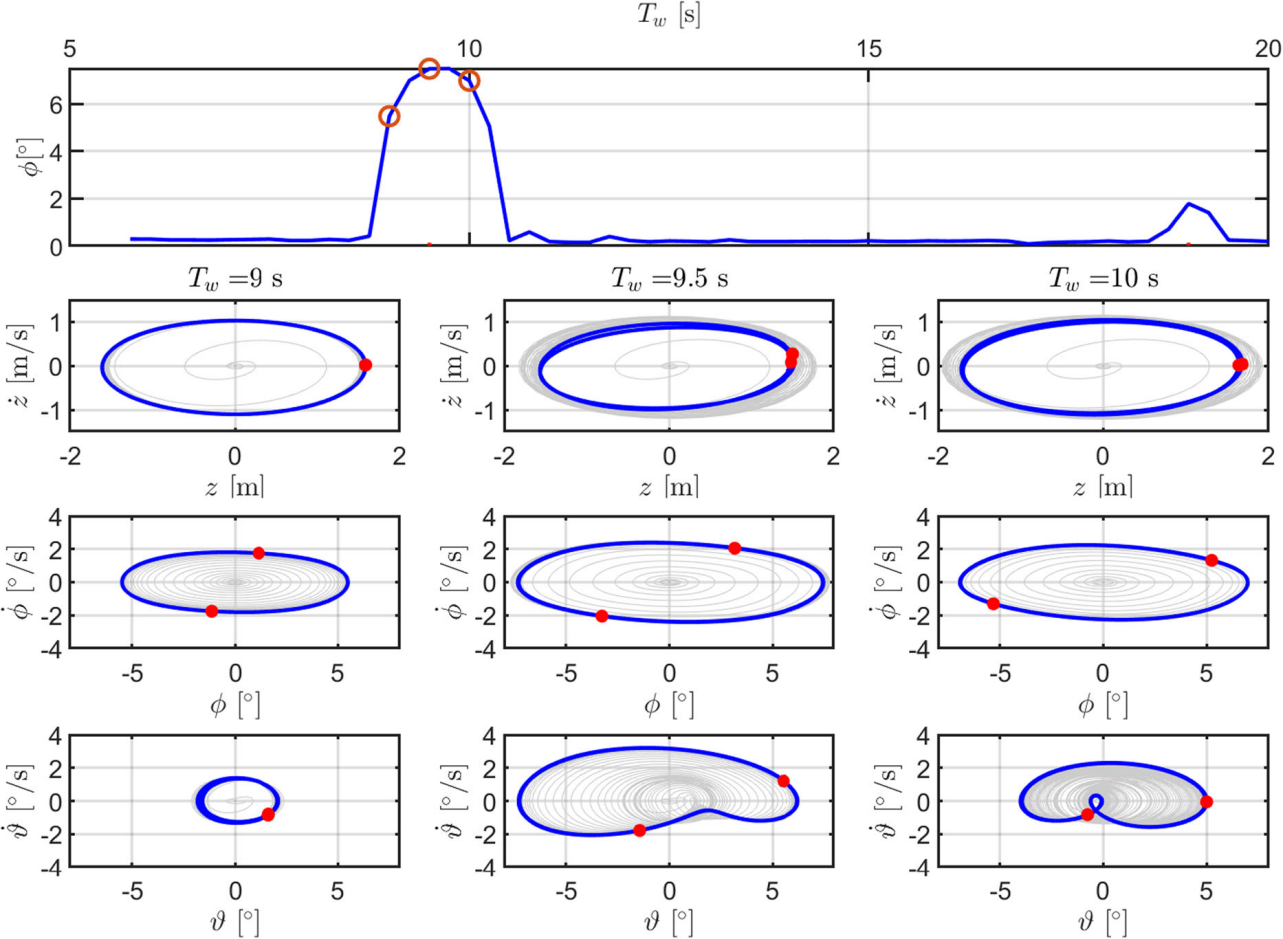
Resonance curve in roll, for configuration DRT4 and $H_w$ of 3 m. Dashed and dash-dotted red lines are at $T_{n,4}/2$ and $T_{n,4}$, respectively. Phase portraits are shown for $T_w$ equal to 9 s (left), 9.5 s (middle), and 10 s (right). The markers in the Poincaré maps are taken at the peaks of the incoming regular wave. (Color figure online)

Further insight in the nonlinear dynamic response of the system can be obtained from the resonance curve in roll, as shown in Fig. 9. For each of three relevant points in the parametric resonance region ($T_w$ equal to 9 s, 9.5 s and 10 s), the phase portraits of heave, roll and pitch are presented in Fig. 9. Moreover, the Poincaré map shows frequency doubling, especially in the rotational DoFs. Note that the markers in the phase portraits are taken at the peaks of the incoming wave.

**Fig. 10:**
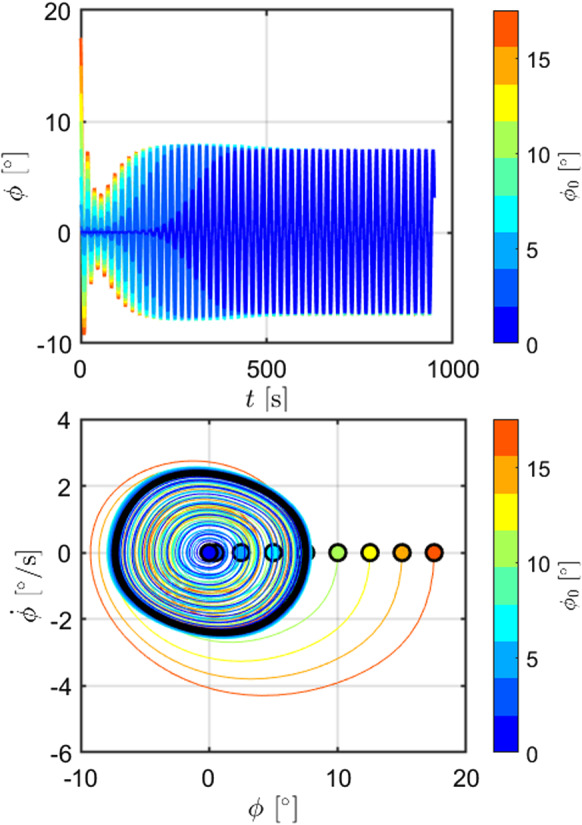
Phase portrait for configuration DRT4, $H_w=3$ m and $T_w=9.5$ s, $d_0=0.864$ m, for 9 different roll initial conditions. The systems show only one attractor, since the same limit cycle is obtained, regardless of the initial condition considered

The influence of changes of initial conditions is studied in Fig. 10, where the phase portraits of a wave in the parametric resonance region ($H_w=3$ m, $T_w=9.5$ s) is studied for 9 different initial conditions ($\phi _0$): one at $\phi _0=0.5^\circ$, and 8 from 0 to $17.5^\circ$, with step equal to $2.5^\circ$. Although such initial conditions span around the steady-state amplitude of the limit cycle, the same attractor is reached. Figure 10 also shows a similar pattern for the transient which, although faster for larger initial conditions, presents the same drop of the envelope after about 55 s of simulations. Furthermore, note that the transient from $\phi _0=0^\circ$ is much longer than the one from $\phi _0=0.5^\circ$. However, since the exact zero in real applications is highly unlikely (if not impossible), in the simulations used to produce all other results, an initial condition of $\phi _0=0.5^\circ$ is assumed, in order to reduce transient periods.

**Fig. 11:**
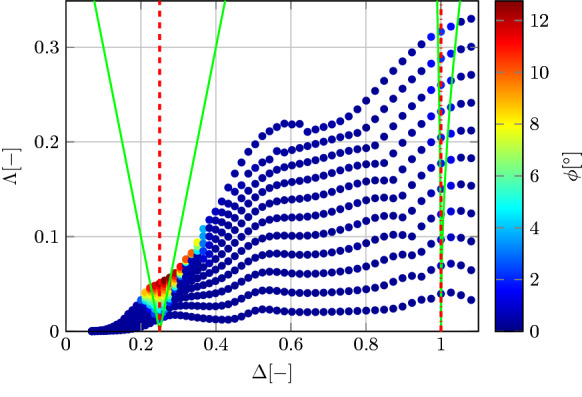
Reconstructed stability diagram from numerical simulation for configuration DRT4 and $d_0=0.864$ m. The green lines show the theoretical limits of stability for $\mu =0$, as in Fig. 1. Maximum non-dimensional damping term ($\mu$) of 0.032. (Color figure online)

Finally, it is worth to reconstruct the stability diagram using the results of the numerical simulations. However, as discussed in Sect. 2, several simplifications are needed to fit the model of (12) into the equation in (2). Let us consider the uncoupled roll DoF and neglect nonlinearities due to the excitation force, kinematics, PTO and mooring systems. Let us consider the linearized definition of hydrostatic stiffness in roll [10]:

16 $$\begin{aligned} K_4 = \rho g \nabla \left( \frac{I_a}{\nabla }-\overline{BG}\right) \end{aligned}$$

where $\rho$ is the water density, *g* the acceleration of gravity, $\nabla$ the submerged volume, $I_a$ the geometrical moment of inertia of the water plane area, and $\overline{BG}$ the distance between centres of buoyancy and gravity. Using the same numerical framework described in Sect. 4.3, the time-varying $\nabla$ and $\overline{BG}$ can be computed according to the 6-DoF displacements [15]. Since the time-variations of $K_4$ are not exactly harmonic, the amplitude $\varLambda$ is estimated as half the excursion from peak to trough of $K_4$ and normalized by its mean. The resulting ($\varDelta -\varLambda$) coordinates are shown in Fig. 11, where the colour of each marker is proportional to the amplitude of the roll response. In this way, the stability diagram can show both regions of instability and the severity of the parametric response.

Consistently with Fig. 1, the main unstable region is located around $\varDelta =0.25$ and widens as $\varLambda$ increases, with a corresponding increase in roll amplitude. A small roll response can be also found at $\varDelta =1$. The reduced extension of this secondary unstable region is due to viscous losses. Note that the area of primary instability is wider than the one predicted by the simplified analytical model (as shown in green in Fig. 11 and in Fig. 1), highlighting the value of using a more representative model of higher complexity for advanced design considerations.

In order to provide a rough comparison with Fig. 1, the non-dimensional linear dissipation coefficient ($\mu$) is defined [11]:

17 $$\begin{aligned} \mu (\omega ) = \frac{B(\omega )+C_{lin}}{\left( I_{x}+A(\omega )\right) \omega } \end{aligned}$$

where *A* is the radiation added mass, *B* is the radiation damping, and $C_{lin}$ is an equivalent linear viscous drag coefficient. $C_{lin}$ is chosen a posteriori such that the resulting linear viscous force dissipates the same energy of the nonlinear force over the same periodic time window [40]. Using the definition in (17), $\mu$, which depends on the incoming wave and motion response, reaches the maximum value of 0.032.

The discussion carried out so far is based on regular waves since, being monochromatic, they are fit to clearly describe the frequency-dependent attitude of the system. However, since real waves are panchromatic stochastic processes, the instability excitation may differ. Ref. [2] studies more realistic non-sinusoidal wave profiles, still inducing instability into the system, while [44] discusses how the instability regions become wider as the noise intensity increases, while tongues of instability domains rise up. This is consistent with experimental observation [23] and numerical modeling [20] of a WEC prototype. Note that the proposed NLFK force calculation can be also applied to irregular wave conditions, as discussed in [20]. Regions of instability become wider because of the spread of spectral energy content across the frequency range. However, due to an unsteady and non-uniform energy supply at the parametric resonance frequency, transients are longer and a sustained instability is reached for a larger overall excitation, namely a larger wave height. Nevertheless, numerical simulations in irregular wave conditions become more sensitive to the representation viscous losses, which have a direct impact on the transient evolution [20].

**Fig. 12:**
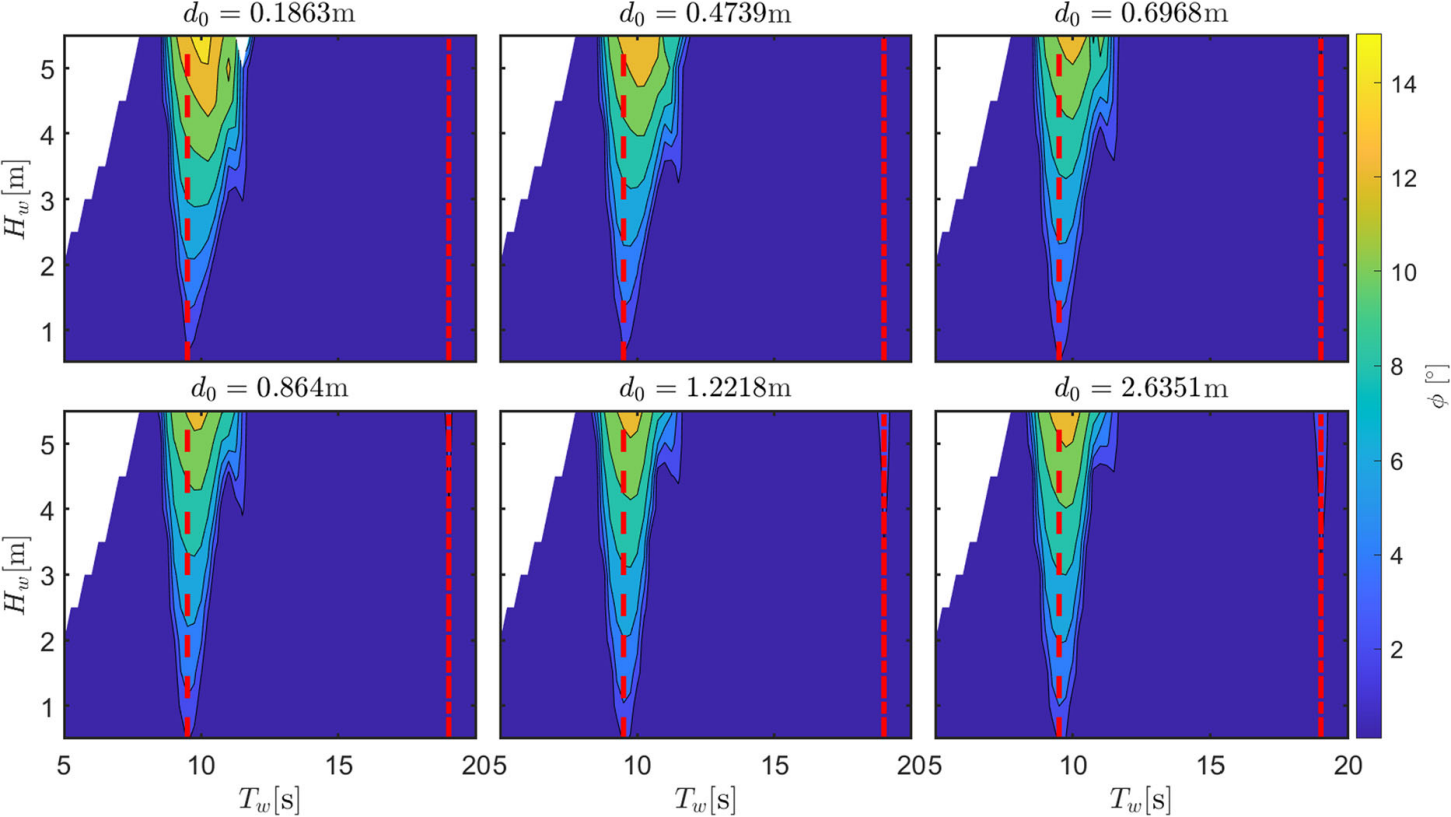
Amplitude of roll response for configuration DRT4 varying the orifice diameter (the smaller $d_0$, the larger the PTO damping). Dashed and dash-dotted red lines are at $T_{n,4}/2$ and $T_{n,4}$, respectively. (Color figure online)

**Fig. 13:**
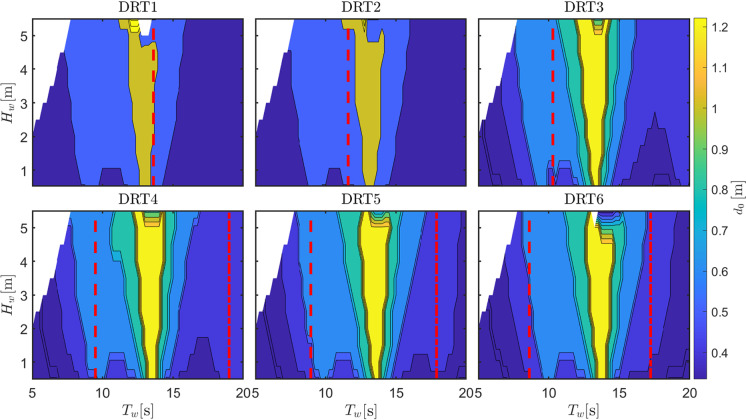
Optimal orifice diameter (for maximum power extraction while avoiding survivability conditions) for different draft configurations

**Fig. 14:**
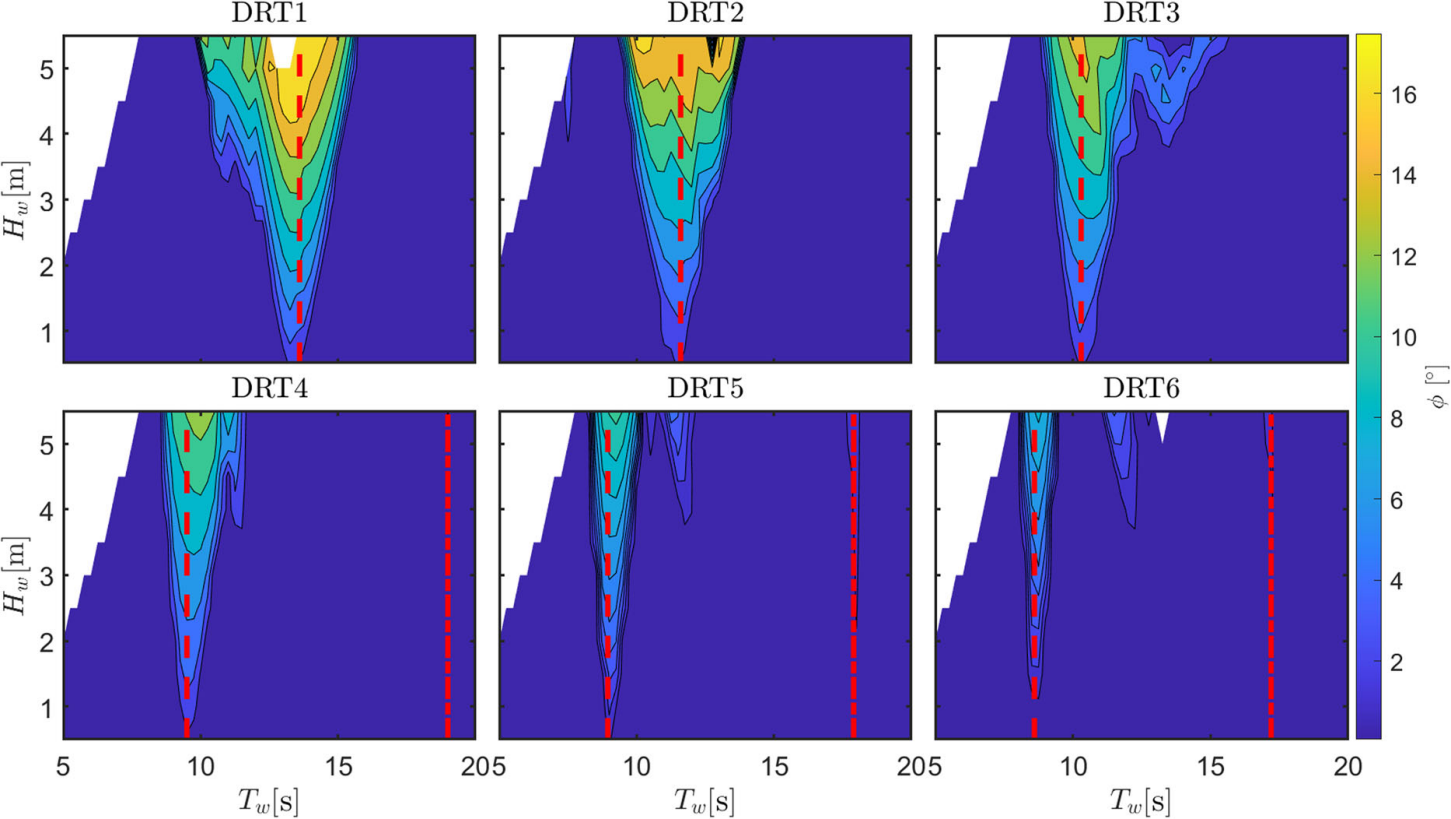
Roll amplitude (using orifice diameters in Fig. 13) for different draft configurations

**Fig. 15:**
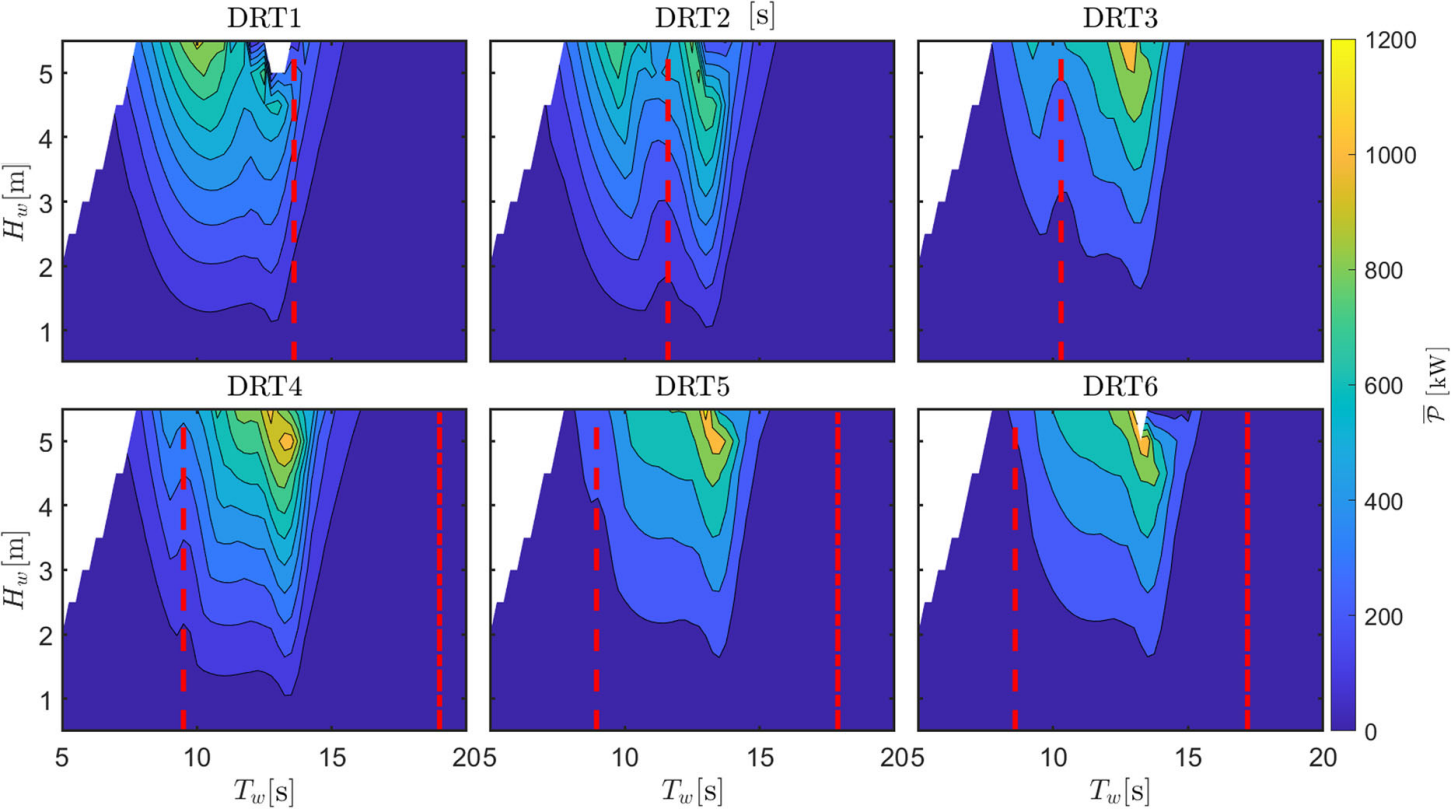
Optimal power extracted (using orifice diameters in Fig. 13) for different draft configurations

### Sensitivity analysis

As discussed in Sect. 2 and shown in Sect. 5, the likelihood of parametric instability mainly depends on the natural period of the parametrically excited DoF, i.e., inertial and restoring properties in that DoF. In addition, the severity of the parametric response also depends on the overall stiffness of the system, as well as internal dissipations. Since the model proposed in this paper is able to quantitatively predict the amplitude of the parametric response, depending on changes of virtually any parameter of the system, it is used to perform a sensitivity analysis that can inform and effectively guide the design of the system.

The first parameter considered is the diameter of the orifice plate, tabulated in Table 3, emulating the damping action of the PTO system. The PTO force, shown in (13), is the control action that is normally used to maximize the absorbed power while remaining compliant to constraints, usually related to the safety of components of the system. In fact, in case of extreme wave conditions, the control strategy should prioritize survivability over power production. A common strategy is to increase the PTO damping in order to avoid relative motions between parts whose relative movement is normally used to extract energy [39]. However, this strategy can be counterproductive if the system is prone to parametric resonance, since a stiffer system tends to experience larger parametric response, potentially threatening the device integrity [39]. This is shown in Fig. 12 for configuration DRT4, where parametric roll increases in amplitude and width as $d_0$ decreases (as the PTO damping increases).

The second relevant parameter considered in the sensitivity analysis is the ballast or, alternatively, the consequent draft. Table 2 shows the 6 different draft configurations, the draft of the top part of the floater, and the consequent natural period in roll, which is the most important parameter determining the region of parametric instability. As the draft increases (due to a higher ballast at the bottom part of the converter), both the hydrostatic stiffness (due to increase in $\overline{BG}$) and the rotational inertia increase. The increase in hydrostatic stiffness presents an higher importance over the inertia variation and, consequently, $T_{n,4}$ decreases. Therefore, parametric instability is expected to appear at lower periods as the draft increases. Figure 13 shows the map of optimal $d_0$ that maximize power production for each wave condition, while ensuring survivability. At a period of about 14 s, all configurations present a relatively large value for the optimum orifice plate diameter. This is associated with an improved excitation of the OWC due to the reduction of the turbine damping effect, since the OWC heave natural period is observed at 14 s. Using $d_0$ from the maps in Fig.  13, Fig. 14 shows the amplitude of parametric response for the different draft configurations.

As expected, the condition for parametric resonance shifts to lower periods as the draft increases. However, the width of the unstable region, as well as the amplitude of response, considerably shrinks as $L_c$ increases. Remarkably, configuration DRT6 is almost unaffected by parametric response, likely due to the increase in the rotational hydrostatic stiffness, which make its relative variation less significant.

Ultimately, since the system is a wave energy converter, the most important quantity to consider is the converted power, as shown in Fig. 15. For all configurations, a clear local drop of power production is visible around the region of parametric instability, confirming the detrimental effect of parametric resonance for all draft configurations. Overall, the best configuration appears to be DRT4, with a wider and higher-power conversion region, as shown in Fig. 15, and a relatively low and localized parametric response, as shown in Fig. 14. From Fig. 15, it seems that configuration DRT6 is the one where the power extraction is less affected by parametric resonance, as the relevant power spectrum falls between the two instability regions at around $T_{n,4}/2$ and $T_{n,4}$. Ultimately, two conflicting design objectives should be balanced, namely power conversion capabilities, shown in Fig. 15, and operability/survivability, which depends on several different aspects, including roll response, shown in Fig. 14. One potential proxy for survivability is the resulting mooring load, as considered in [16], or the maximum pitch/roll angle, which may affect the structural integrity of the tube.

As a final remark, note that all discussion and sensitivity analysis herein performed is based on idealized monochromatic waves, which are simple and concise, carrying univocal frequency and amplitude information. However, real waves are random realization of a stochastic process, so the likelihood and severity of parametric resonance, although correlated to regular wave conditions, is potentially changing. Therefore, consideration regarding relative advantages of different design solutions is to be read as preliminary and further investigation and testing is required to corroborate such conclusions.

## Conclusions

Although parametric instability is a common nonlinear phenomenon for spar-like floating structures, it is not often included in the design process. In fact, mathematical models, essential to inform the design stage, usually are either oversimplifying the system, or are computationally too slow. On the one hand, the vast majority of analytical models used to study parametric resonance are developed in two degrees of freedom, idealize the geometry and neglect the interaction with other phenomena or ancillary systems (such as nonlinear excitation, power take-off and mooring systems). Furthermore, while all assess well the likelihood of parametric instability, further assumptions are usually needed to predict the severity of the parametric response. On the other hand, conventional nonlinear time-domain models, although more representative of the full complexity of the system, are usually too time-consuming to be used for extensive sensitivity analysis.

The model proposed in this paper purports to bridge such a gap. A computationally efficient model for floating spars is presented, considering a realistic wave energy converter as a case study, including nonlinear kinematics, an analytical formulation of nonlinear Froude–Krylov forces, viscous drag forces, PTO and a realistic mooring system. Such a model is able to quantitatively articulate parametric resonance, showing its detrimental effects on power extraction efficiency. The model is also used to reconstruct the stability diagram of the system, based on numerical simulations, which is compared with predictions from the simplified Mathieu equation. The numerical model is then used to study the sensitivity to the control force and to the ballast configuration, determining the best option that limits parametric roll while optimizing power extraction.
